# Acute Angle-Closure Glaucoma Secondary to a Phakic Intraocular Lens, an Ophthalmic Emergency

**DOI:** 10.5811/cpcem.2019.1.41399

**Published:** 2019-03-05

**Authors:** Arian Frost, Daniel J. Ritter, Alana Trotter, Michael S. Pulia

**Affiliations:** * University of Wisconsin-Madison, School of Medicine and Public Health, Madison, Wisconsin; † University of Wisconsin-Madison, School of Medicine and Public Health, BerbeeWalsh Department of Emergency Medicine, Madison, Wisconsin; ‡ University of Wisconsin-Madison, School of Medicine and Public Health, Department of Ophthalmology and Visual Sciences, Madison, Wisconsin

## Abstract

Implantable collamer lenses (ICL) are phakic (natural lens remains in place) lenses that were first developed in the 1990s for correction of high myopia. The effectiveness and safety of ICLs are making them an increasingly popular option for vision correction in the myopic patient, competing with traditional options like glasses, contacts, and procedures such as laser-assisted in situ keratomileusis. Although generally safe, due to the position of the phakic ICL in the eye, pupillary block remains a rare but vision-threatening complication of ICL implantation. Pupillary block caused by phakic ICL is a serious complication that requires urgent recognition and intervention and is poorly described in emergency medicine literature. We describe a case of pupillary block five years after ICL implantation that was refractory to standard medical therapy, highlighting the importance of early diagnosis and referral for more definitive therapy.

## INTRODUCTION

Implantable collamer lenses (ICL) are specialized refractive intraocular lenses used to correct high myopia. Made of specialized collagen copolymer, phakic ICLs are surgically implanted inside the eye, sitting between the iris and the natural lens. These artificial lenses were first implanted in 1993 and approved by the United States (U.S.) Food and Drug Administration in 2005. They have since undergone multiple revisions to minimize complications and to increase utilization and potential indications.^1^ When they were first released, ICLs were commonly used for high and extreme myopia. Low and moderate myopia were primarily treated with procedures such as laser-assisted in situ keratomileusis (LASIK), which is a permanent solution not available to patients with dry eye or thin cornea. Studies have compared the two treatments and made a case that ICLs are more effective and safer in the treatment of all cases of myopia.^1^,^2^

Although relatively safe, ICLs are placed in the ciliary sulcus, and without adequate pre-operative measurements they carry the risk of pupillary block, inflammation, cataract formation, and intraocular hypertension. Overall, less than 1% of patients with ICLs experience serious, vision-threatening complications.^3^ These complications, however, are most commonly observed in the immediate and subacute postoperative period. A remote increase in intraocular pressure and pupillary block secondary to ICL implantation is not well documented. We present a case of acute angle-closure glaucoma secondary to pupillary block due to mechanical obstruction from an ICL five years after implantation.

## CASE REPORT

A 29-year-old woman with high myopia and a history of bilateral ICLs placed five years previously presented to the emergency department (ED) with a chief complaint of headache and blurry vision in her right eye. The patient stated that the night prior to presentation she noted that her right eye was dilated. She also complained of light sensitivity and mild blurry vision. When she woke up the morning of presentation she noted a dull headache behind her right eye, which she rated a 2/10 on a numeric pain scale. She reported trouble focusing on close-up text but denied other vision decline or diplopia. She denied neck pain, nausea, vomiting, fever, chills, numbness, or tingling. She denied recent trauma, visits to the chiropractor, or use of mydriatic medications. She had been evaluated by ophthalmology six days prior to presentation for similar symptoms and was found to have mild mechanical anisocoria. Given her minor symptoms at that time, it was felt that there was no need for intervention.

Initial examination in the ED revealed a marked anisocoria, with the right pupil larger than the left. The right pupil was mid-dilated and fixed at six millimeters (mm). There was appropriate constriction of the left pupil. The right conjunctiva was injected. Visual acuity was 20/30 in the right eye and 20/20 in the left eye. Intraocular pressure of the right eye was markedly elevated at 44 mm of mercury (Hg). Her remaining neurologic exam revealed no focal deficits. Ophthalmology was consulted. After examining her, they found a right eye with a round, fixed pupil, +1 injection, diffuse microcysts, a shallow anterior chamber, fixed, minor iris bombe, and confirmation of intraocular hypertension. Examination of the left eye demonstrated two peripheral patent iridotomies at 12 o’clock and 3 o’clock and intraocular pressure of 11 mmHg. The patient was diagnosed with acute pupillary block and was administered timolol, acetazolamide, and brimonidine, but the intraocular pressure remained elevated at 35 mmHg. The patient was discharged from the ED directly to an outpatient ophthalmology clinic for urgent procedural treatment of pupillary block.

The patient presented to the ophthalmology clinic and underwent a yttrium-aluminum-garnet laser peripheral iridotomy. The right eye was anesthetized with topical proparacaine and a single peripheral iridotomy was created in the temporal iris. Aqueous humor was visualized to flow through the ostomy from the posterior to anterior chamber and the iris bombe significantly flattened. Immediately post-procedure, topical brimonidine was administered and the intraocular pressure was measured at 17 mmHg. One hour after the procedure, the intraocular pressure was 13 mmHg. The patient was prescribed difluprednate four times daily and brimonidine/timolol twice daily for five days and scheduled for follow-up with ophthalmology in one week.

At one-week follow-up, the patient noted marked improvement of her symptoms. She did not note any further headaches or difficulty with vision. She did, however, note continued mild anisocoria in the dark, and examination confirmed 0.5–1mm right greater than left anisocoria. Due to the persistent anisocoria, the patient was referred to neuro-ophthalmology for further investigation. A neurologic etiology was not identified and the persistent anisocoria was felt to be mechanical. The patient returned to ophthalmology clinic several months after her initial presentation with a subsequent increase in her intraocular pressure and required an additional peripheral iridotomy. She continues to be followed by ophthalmology.

**CPC-EM Capsule**

What do we already know about this clinical entity?

*Acute angle-closure glaucoma is a vision-threatening process that must be rapidly identified by emergency care providers*.

What makes this presentation of disease reportable?

*This report highlights an unusual case of acute angle-closure glaucoma secondary to a phakic intraocular lens that could have been missed without awareness of this potential complication*.

What is the major learning point?

*Acute angle-closure glaucoma is a potential complication of phakic intraocular lens implantation, which is increasingly being used to correct myopia*.

How might this improve emergency medicine practice?

*This report seeks to provide a diagnostic and therapeutic approach to an unusual case of acute angle-closure glaucoma due to phakic intraocular lens implantation*.

## DISCUSSION

No matter its cause, acute angle-closure glaucoma is an ophthalmological emergency that often requires a high degree of suspicion from the emergency physician. Patients with acute angle-closure glaucoma often have onset of symptoms in low-light conditions due to pupillary dilation resulting in apposition of the iris and lens. This contact is termed pupillary block and results in obstruction of aqueous flow from the posterior chamber. The restricted aqueous flow generates pressure that displaces the iris forward (bombe), narrowing the anterior chamber angle and restricting aqueous drainage through the trabecular meshwork and Schlemm’s canal. The resulting elevation of intraocular pressure causes symptoms that include blurry vision with halos around bright objects, headache, and nausea and vomiting. Physical findings are typically unilateral and include an injected eye with a non-reactive and dilated pupil, and corneal haziness.^4^ Any of these symptoms warrants at least consideration of acute angle-closure glaucoma in the ED.

Angle-closure glaucoma requires prompt diagnosis and treatment to prevent irreversible vision loss. Complete examination of ocular complaints should always include measurement of intraocular pressure. A generally accepted range for normal intraocular pressure is 8–21 mmHg, whereas acute angle-closure glaucoma will have intraocular pressure over 30 mmHg.^5^ Medical therapy is aimed at relieving pupillary block and reducing additional aqueous humor production. These aims are initially achieved with a combination of a systemic carbonic anhydrase inhibitor as well as topical agents including steroids, beta-blockers, and alpha agonists. After one hour of treatment, topical pilocarpine can be added to constrict the pupil (miotic) and relieve the block. Keeping the patient supine and aggressively managing symptoms (pain, nausea and vomiting) to avoid spikes in intraocular pressure are also critical management steps. Definitive management of acute angle-closure glaucoma includes emergent referral for ophthalmologic evaluation and consideration of surgical iridotomy.^6^,^7^

Acute pupillary block is a rare but serious vision-threatening complication of ICLs and is not well reported in the emergency medicine literature. Additionally, much of what is reported in the literature focuses on cases in the immediate postoperative timeframe.^3^,^8^,^9^ Evidence suggests the rate of pupillary block two years after ICL implantation was found to be between 0.1–3.2%.^10^,^11^ Medical management of increased intraocular pressure serves as an attempted bridge to lower the intraocular pressure in preparation for an iridotomy or other definitive surgical management.

## CONCLUSION

The case presented describes an unusual complication of an ICL, delayed pupillary block and acute glaucoma five years after implantation. Given the increasing utilization of ICLs, emergency physicians should become familiar with this known complication to enhance early detection and emergent ophthalmology consultation for definitive management.
